# Applications of Advanced Electrospun Nanofibrous Materials in Water Treatment

**DOI:** 10.3390/nano15181424

**Published:** 2025-09-16

**Authors:** Aiqi Wang, Rui Pan, Jinlei Miao, Yishen Lin, Yuanrou Deng, Siqi Peng, Xiuhuan Luo, Pinhong Wu, Shuting Zhang, Yuanyuan Wu, Jiarong Xie, Tingting Fan

**Affiliations:** 1 Faculty of Pharmacy and Laboratory, Huizhou Health Sciences Polytechnic, Huizhou 516025, China; wangaiqi2019@163.com (A.W.); panruibb@163.com (R.P.);; 2 Industrial Research Institute of Nonwovens & Technical Textiles, Shandong Center for Engineered Nonwovens, College of Textiles & Clothing, Qingdao University, Qingdao 266071, China; 3 Department of Nursing, Huizhou Health Sciences Polytechnic, Huizhou 516025, China

**Keywords:** electrospinning, nanofiber, membrane distillation, oil–water separation, solar evaporation

## Abstract

With the rapid development of cities and increasingly serious environmental problems, energy conservation and environmental restoration are particularly important. Due to the high efficiency of membrane technology in wastewater treatment and polluted water recovery, its research has increased exponentially in recent years. Electrospinning technology has been widely used by researchers in the field of nanofibrous membrane manufacturing due to its inherently high porosity, high flux, and easy preparation of various materials. In this review, the application progress of electrospun nanofibrous materials in water treatment is presented, including membrane distillation, oil–water separation, solar evaporation, and water pollutant removal. Moreover, this review also summarizes the structural design and preparation method of these nanofibrous materials in detail. Finally, the challenges and application prospects of electrospinning in water treatment are determined.

## 1. Introduction

With the development of industrialization, water pollution is becoming more and more serious, and freshwater resources are very scarce [1,2]. So far, there is no safe drinking water in the world, especially in places like Africa [3]. Seawater pollution, industrial wastewater, and domestic sewage discharge are the important factors that cause water pollution [4,5,6]. Increasingly serious water pollution has affected the ecological environment and human health [7]. Traditional water treatment methods, such as filtration [8,9], adsorption [10], and flocculation [11], often have the disadvantages of low efficiency, high energy consumption, and high pollution. New and emerging technologies have been developed to meet the huge demand for freshwater and to address environmental challenges to create more efficient and reliable wastewater treatment methods. In recent years, the excellent properties and unique microstructure of nanomaterials have attracted people’s attention. Electrospinning is a simple and universal technique for the preparation of nanomaterials. The electrospinning device is mainly composed of a propulsion pump, a syringe, a high-voltage power supply, and a receiving device [12]. Polymer solution or melt is placed in a high-voltage electric field; the droplets at the tip of the needle change from spherical to tapered (Taylor cone) and extend from the tip to the membrane filaments, which are then accepted by the collector [13]. By the electrostatic repulsion between surface charges, the nano-spinneret can obtain continuous nanomembranes from viscoelastic fluids [14]. The parameter setting of the spinning machine and the environmental condition are important factors that affect the membrane morphology [15]. Electrospinning technology can arrange, fold, and assemble the membranes into ordered or layered structures to form the nanomembrane with high porosity, large specific surface area, and interconnected pore structure [1,16,17,18].

**Figure 1:**
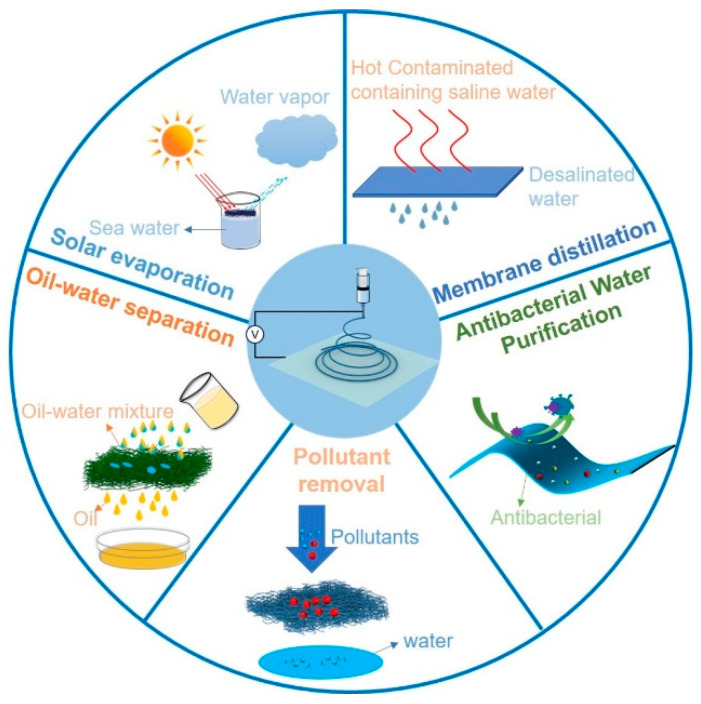
Overview of recent application of electrospinning nanofibrous materials in water treatment: membrane distillation, oil/water separation, solar evaporation, pollutant removal, and antibacterial water purification.

Due to its inherent advantages, electrospinning is widely used in water purification [19], tissue engineering, regenerative medicine [20], smart textiles [21], sensors [22], energy [23], and other fields. Multifunctional 2D nanomembranes and 3D porous materials can be prepared by electrospinning combined with polymer treatment or interfacial modification [24,25]. The functionalized nanomembrane has high mechano-chemical stability and flexibility for efficient water treatment [26,27,28]. This review primarily focuses on the research status and recent advancements in advanced electrospun nanofiber materials for water treatment. It specifically highlights their practical applications in membrane distillation, oil–water separation, solar-driven evaporation, and pollutant removal (Figure 1). The article begins by introducing the micro- and nanostructural advantages of electrospun nanofiber membranes, followed by an analysis of the factors influencing their preparation. Subsequently, the selection of materials for electrospun membranes and their modification methods are discussed. Furthermore, this review addresses the advantages of nanofiber membranes in water treatment, current technical bottlenecks, and future research directions, emphasizing their promising potential for broad application prospects in the water treatment field.

## 2. Membrane Distillation

Membrane distillation (MD) is a process in which the vapor pressure difference on both sides of the membrane is used as the driving force and a porous hydrophobic separation membrane is used for membrane separation. For MD in the separation process, a diffusion of vapor molecules move from the feed out through the porous hydrophobic structure and are finally condensed [29] on the permeate side. Therefore, the separation process is not restricted by the thermodynamic equilibrium, and the theoretical rejection rate for solutes of different concentrations of nonvolatile matter reaches 100% [30]. Because of these advantages, compared with other membrane separations, it has better effects in solar evaporation and sewage treatment [31]. However, an MD separation membrane with excellent performance must meet the following factors: (1): The membrane should have high porosity, low tortuosity, and appropriate pore size and pore size distribution to allow water vapor to pass through the membrane pores with low resistance [32]. (2): The membrane material should have two elements: One is that the membrane material must be hydrophobic. The purpose is to prevent the intrusion of water from the feed side into the permeate side while ensuring a high salt rejection rate. The second is that it must have a certain degree of heat resistance to ensure the thermal stability of the membrane and the stability of the membrane pore structure under high-temperature conditions.

### 2.1. Problems and Solutions for Membrane Distillation

However, the current membrane distillation technology is limited by the problems of low membrane distillation flux and high processing costs. So, the current discussion on how to develop a high steam flux is the focus of research in this field. Traditionally, many processes such as phase inversion, stretching, sintering, or thermally induced phase separation are used for membrane preparation [33,34]. However, the resulting membranes all face a series of problems, such as low permeability, low hydrophobicity, pollution and wetting tendencies, and so on. Therefore, to meet the requirements of the membrane distillation process, it is necessary to explore new membranes to improve the performance of MD. As a special fiber manufacturing process, electrospinning can produce micro-nanoporous structures with high porosity. Compared to traditional membrane materials, electrospun nanomembranes have higher porosity. In electrospinning technology, a strong electric field is usually applied. When the electrostatic force overcomes the surface tension under the action of the electric field, the polymer solution is usually stretched in a complicated path [35]. Under conditions where the solvent evaporates quickly, the nanofibers are left on the receiver to form a fibrous membrane. At present, electrospun nanofiber membranes have been successfully applied to ultrafiltration [36] and nanofiltration [37]. With the continuous maturity of membrane distillation technology, electrospun nanofiber membranes are gradually applied to the field of membrane distillation. At the same time, they exhibit excellent distillation performance due to the characteristics of electrospinning. However, high-flux nanofiber membranes also face many problems, such as the deterioration of the mechanical stability of the membrane and membrane fouling. This article reviews the preparation of distillation membranes, the structure and modification of membranes, and the application of Janus membranes and distillation membranes.

### 2.2. Preparation of Membranes

Electrospinning is used as a micro-nanofiber manufacturing process. Since the early 1990s, significant progress has been made in controlling the formation of nanofibers, which has promoted the development of nanotechnology and the emergence of modern analytical methods [38,39]. Compared to other methods, electrospinning with lower cost and higher productivity has full advantages in fiber manufacturing [40,41]. The electrospinning process is mainly composed of three important parts: the high-voltage electric field power supply, the injector, and the grounded collection device. For obtaining nanofiber membranes with different porosities, fiber diameters, and thicknesses, it is possible by changing the process parameters or the properties of the spinning solution. This work summarizes the electrospinning parameters of some reference materials in Table 1.

### 2.3. Factors

In this part, we mainly discuss the properties of MD membranes by discussing the spinning process parameters and the properties of the spinning solution. It should be noted that these factors will mainly affect the flux of the distillation membrane. In theory, the retention of pollutants by the MD membrane will still be close to 100%.

#### 2.3.1. Operational Characteristics

It is well known that the structure and morphology of nanofiber membranes depend to a large extent on the operating conditions of electrospinning. Operation may affect the generation of MD membranes, including the size of the applied electric field, the head and the distance between the stations, temperature, humidity, and the spinning time discussions accepted. One of the key parameters is the magnitude of the applied electric field. The magnitude of the applied electric field plays a decisive role in the magnitude of the electric field force received by the nozzle [42]. Large electric field forces will produce thinner fiber membranes. The distance between the tip of the spinneret and the surface of the collecting device is called the receiving distance. Since the solidification of nanofibers depends on the rapid volatilization of the solvent, a complete fiber will not be obtained for a short distance, and a humid environment will aggravate this phenomenon and form a porous structure [43]. Regarding the spinning time, a positive relationship is formed between the obtained nanofiber membranes by adjusting the electrospinning time. Finally, for MD membranes, the thickness of the membrane is a very important influencing factor for the heat and mass transfer process [44].

#### 2.3.2. Spinning Solution Properties

In this section, some key parameters will be discussed, including polymer concentration, molecular weight, conductivity, surface tension, and solvent selection. As we all know, the properties of nanofibers produced by electrospinning are mainly affected by the composition of the polymer [45,46,47,48]. For electrospinning, every pair of polymers and solvents exists to successfully to know the maximum concentration of nanofiber polymers. A suitable concentration ratio will help to obtain nanofibers with a uniform cylindrical structure. An improper concentration ratio may cause the fiber diameter to increase or it may not be able to spin [49]. At the same time, the molecular weight of the polymer will affect the viscosity of the spinning solution and thus affect the electrical properties. Finally, the size of the molecular weight will affect the polymer concentration ratio. The electrical conductivity of a polymer is affected by the properties of the polymer, the properties of the solvent, and the presence of ionizable particles [50]. The electrical conductivity of the fiber has an impact on the final nanofiber structure. The high conductivity of the spinning solution means that the charge capacity increases, and it will expand more fully under a specific electrostatic field [51]. Therefore, a highly conductive solution is preferred in electrospinning, and we can increase the conductivity by introducing conductive particles into the spinning solution. However, excessively high conductivity will form an unstable Taylor cone and affect the properties of the fabric [52]. In this case, the surface tension of the spinning solution becomes particularly important. The surface tension of the spinning solution can be adjusted by adjusting the composition of the solution to optimize the fiber membrane structure and dream distillation performance [53,54].

### 2.4. Selection and Modification of Membrane Materials

The separation membrane used in membrane distillation technology must meet the following criteria: (1): hydrophobicity and (2): porosity. This is a requirement for membrane material and membrane structure. At the same time, due to the electrospinning process requirements, the material must have good membrane-forming properties. If the membrane material has high thermal resistance, thermal stability, and pore structure stability based on hydrophobicity, it will have strong development potential in the field of membrane distillation. In addition, the hydrophobicity or mechanical strength of the MD membrane can be enhanced by modifying the membrane material.

#### 2.4.1. Material Selection of Membrane

Due to the technical requirements of membrane distillation, the traditional hydrophobic membrane materials for membrane distillation are mainly polyvinylidene fluoride (PVDF) [55], polytetrafluoroethylene (PTFE) [56], polyethylene (PP) [57], and polyethylene [58,59]. Among them, PVDF has long been used to make various separation membranes. For example, PVDF has long been used to make drinking water from salt water [60]. This is because it has excellent solubility, mechanical strength, thermal stability, and chemical stability and is often used in electrospinning processes. Therefore, PVDF is one of the most ideal materials for distillation membranes. At the same time, compared to the traditional membrane-making method, the water contact angle of the electrospun nanofiber membrane has been significantly improved, which improves the hydrophobic performance. At the same time, because the electrospinning adopts the method of overlapping fibers to form a membrane, the flux of the PVDF membrane will also be significantly improved when it is used for membrane distillation. To further improve the hydrophobicity of membrane materials, some PVDF copolymers such as PVDF copolymerized hexafluoropropylene (PVDF-HFP) have been widely used and successfully applied to the preparation of MD membranes [61].

#### 2.4.2. Modification of the Membrane

At present, the main material for membrane distillation is PVDF. Therefore, in addition to the development of other membrane materials that can be used for electrospinning, modification of existing nanofibers has also become one of the effective ways to prepare membranes used in the MD process.

#### 2.4.3. Inorganic Nanoparticle Modification

**Figure 2:**
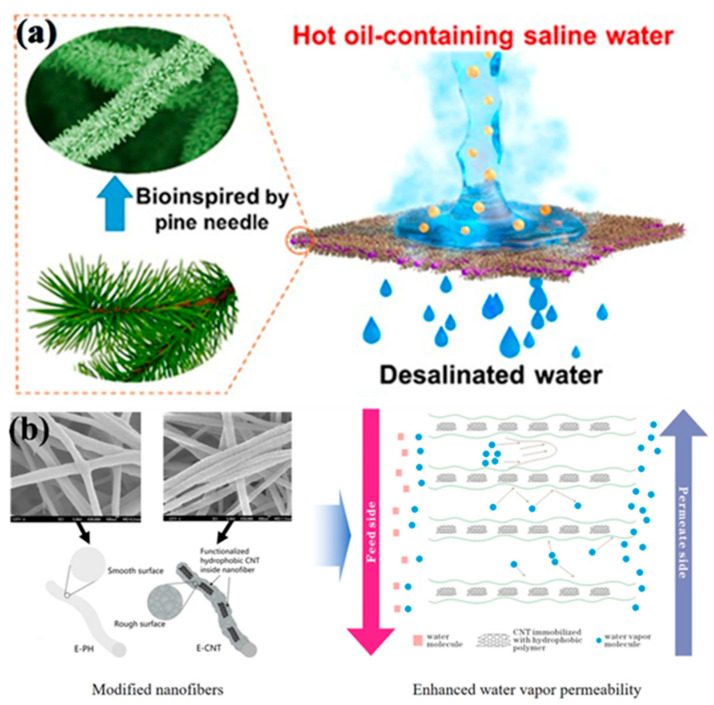
(**a**) Nano-titanium dioxide grows on the surface of the fiber to form a pine needle makeup structure and is used for membrane distillation. Reprinted from [64] with open access license. (**b**) The CNT composite electrospun membrane exhibited enhanced vapor transport. Reprinted from [65] with open access license.

To obtain the modified electrospun nanofibrous membrane, some nanoparticles are incorporated into the polymer spinning solution. Conventional nanoparticles are introduced into two main categories: one is hydrophobic nanoparticles, such as nano-SiO_2_, nano-Al_2_O_3_ and so on. The addition of SiO_2_ nanoparticles can make nanofiber membranes form superhydrophobic surfaces by adjusting the surface roughness [62]. In addition, nano-Al_2_O_3_ hydrophobized with isostearic acid [63] can also effectively improve the surface hydrophobicity of nanofiber membranes and membrane distillation flux. Another method is to incorporate hydrophilic inorganic nanoparticles such as TiO_2_ and CNTs. Due to its unique characteristics, titanium dioxide is one of the most widely used inorganic nanoparticles [64]. These nanoparticles can effectively improve the hydrophobicity of the fiber membrane surface and the membrane’s distilled water flux. In addition, since the incorporated nanoparticles are modified by blending, the hydrophobicity inside the membrane pores will also be enhanced. At the same time, water vapor will quickly pass through the pores in the form of viscous flow due to the diffusion of molecules. Finally, in terms of heat transfer efficiency, the baffle effect formed by the protrusion of the nanoparticles will reduce the heat conduction effect and improve the heat transfer efficiency. Also, as shown in Figure 2a, titanium dioxide nanorods are grown on polyvinylidene fluoride–hexafluoropropylene (PVDF-HFP) nanofibers by the hydrothermal method, and the titanium dioxide is grown on the fibers in a uniform pine needle shape to make a fully hydrophobic membrane. The fiber membranes manufactured by this method have contact angles of 168° and 153° to water and mineral oil, respectively, and both exhibit superhydrophobic properties. And it can be guaranteed that the high stability of the 99.99% salt interception performance can be used to recover water from industrial wastewater. The prepared carbon nanotube (CNT) composite electrospun membrane exhibited higher flux than the pure poly(vinylidene fluoride-co-hexafluoropropylene) electrospun membrane in the membrane desalination tests, as shown in Figure 2b [65].

Furthermore, the role of protruding nanoparticles in heat transport is twofold. On the one hand, excessive protrusion may weaken the contact between adjacent fibers and introduce interfacial voids, thus limiting the efficiency of solid–solid thermal conduction within the membrane. On the other hand, exposed nanoparticles enhance surface light absorption and local photothermal conversion. In addition, the intrinsic thermal conductivity of nanoparticles such as CNTs and Fe_3_O_4_ can facilitate the formation of thermally conductive pathways throughout the fibrous matrix. Therefore, both the geometric morphology and thermal properties of the nanoparticles must be considered in evaluating their overall impact on heat transfer performance.

#### 2.4.4. Surface Spray Modification

**Figure 3:**
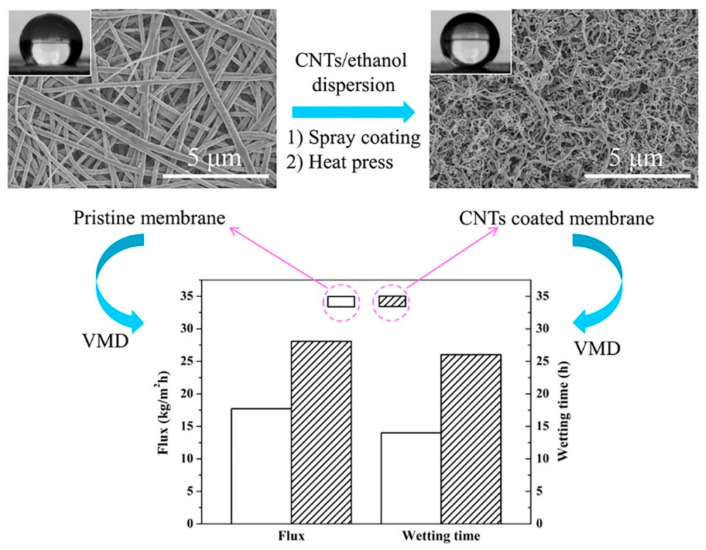
The carbon nanotube fiber coating surface SEM image under conditions of ethanol as a solvent. Reprinted from [67] with open access license.

Carbon nanotubes are an ideal hydrophobic material and are widely used for hydrophobic modification of thin membranes. For example, carbon nanotubes are introduced into conventional polymer spinning solutions to enhance hydrophobic properties [66]. Compared to commercial polyvinylidene fluoride (PVDF) membranes, nanocomposites containing carbon nanotubes will have a higher water contact angle [65]. However, due to the characteristics of carbon nanotubes, it is difficult to completely disperse them water. Therefore, the problem of carbon nanotube agglomeration in the polymerization solution will affect the electrospinning process and the entire membrane-forming process. Therefore, for carbon nanotubes, maintaining a low content of a suitable solvent can have a sufficient effect on the agglomeration of carbon nanotubes. Then choosing the spraying method can change the properties of the membrane surface [67]. As shown in the electron micrograph of Figure 3, it can be seen that under the condition of ethanol as the solvent, the carbon nanotubes do not aggregate into large clusters but are evenly dispersed on the surface of the membrane.

#### 2.4.5. Membrane Surface Chemical Graft

Membrane surface grafting refers to the chemical reaction on the membrane surface through various methods which is used to graft some hydrophobic groups to the surface of the fiber membrane. For example, surface grafting is carried out by physical methods through CF_4_ plasma irradiation technology [68]. The hydrophobic nanoparticle octavinyl graft polyhedral oligosiloxane was introduced into polytetrafluoroethylene to enhance the mechanical strength and porosity of the membrane [69]. Due to its excellent performance, the water contact angle of the optimized PTFE membrane rose to 151 ± 4°, and at the same time, it exhibited a strong membrane flux.

#### 2.4.6. Janus Membrane

**Figure 4:**
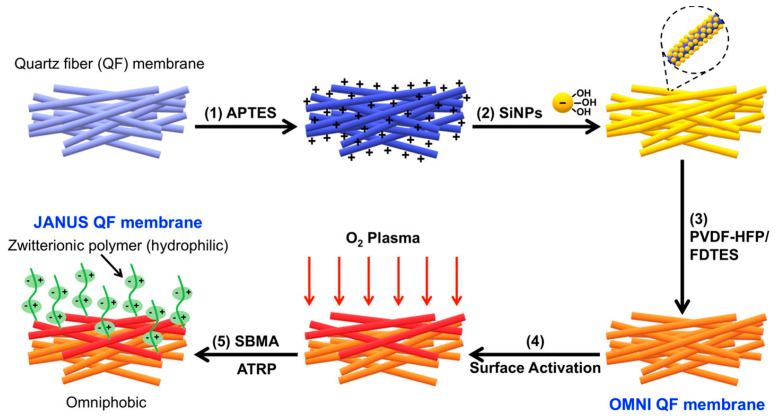
Schematic illustration of the Omniphobic (OMNI QF) membrane fabrication and Janus (JANUS QF) membrane fabrication by grafting a zwitterionic polymer brush layer on the Omniphobic substrate (OMNI QF). Reprinted from [72] with open access license.

Janus membrane, a membrane consisting of an asymmetric structure, now generally receives unprecedented attention due to its unique characteristics [70,71]. For example, as shown in Figure 4, a Janus membrane has been developed to have both antifouling and wetting properties [72]. At present, Janus membranes in the form of flat and hollow fibers are used in membrane distillation [32,73,74,75]. If the electrospun nanofiber membrane is combined with another electrospun nanomembrane or a traditional separation membrane, the electrospun nano Janus membrane used for membrane distillation can be obtained. For example, by adjusting the spinning time of different components, the hydrophobic PVDF and hydrophilic polyethersulfone (PES) solutions are spun into a hydrophobic and hydrophilic two-layer membrane. The PVDF in the Janus membrane is hydrophobic. The relatively thin layer facilitates separation. A thicker PES hydrophilic layer is beneficial to improve the quality and express benefits while enhancing the mechanical strength, or to combine the hydrophilic, high-porosity, and porous nanofiber membrane made by electrospinning with the traditional phase separation membrane with small pores. The influent hydrophobic Janus membrane is made to effectively improve the distillation performance of the membrane.

The vapor transport behavior through electrospun membranes is highly dependent on pore size distribution. In particular, when the characteristic pore diameter is small, Knudsen diffusion dominates, wherein gas molecules collide more frequently with pore walls than with each other. Conversely, in membranes with larger pore sizes, viscous flow becomes the primary transport mechanism. Therefore, tuning the pore structure is critical for optimizing membrane distillation performance. In electrospinning, pore size can be indirectly controlled by adjusting parameters such as polymer concentration, solution viscosity, applied voltage, and collector distance. For example, lower polymer concentration and higher voltage typically result in thinner fibers and smaller pore sizes. These process–property relationships enable the fabrication of membranes tailored for specific transport regimes. Francis et al. [76] demonstrated that post-fabrication treatments—including CNT electrospraying and heat pressing—can reduce the mean pore size and narrow the pore-size distribution of PVDF-Co-HFP electrospun membranes by ~36%, resulting in increased liquid entry pressure and enhanced vapor flux stability in membrane distillation. These findings highlight the practical impact of pore structure engineering on transmission regimes and membrane performance.

The structure–property relationships of electrospun membranes play a critical role in determining their distillation performance. Surface modifications, such as the incorporation of nanoparticles (e.g., SiO_2_, TiO_2_) or hydrophobic functional layers, can effectively improve the surface roughness and reduce surface energy, thereby enhancing the hydrophobicity and anti-wetting capacity of the membrane. In addition, fiber diameter, pore size distribution, porosity, and membrane thickness directly affect vapor permeability, thermal conductivity, and mechanical strength. Through rational material selection and structural optimization, electrospun nanofibrous membranes can achieve high water vapor flux, stable salt rejection, and excellent thermal and mechanical stability in membrane distillation applications.

### 2.5. Application

The structural and surface characteristics of electrospun membranes not only govern their intrinsic properties but also exert direct influence on their practical performance in membrane distillation. Specifically, hierarchical pore structures, asymmetric wettability, and multi-layer architectures can tailor the pathways for water vapor transport, enhance thermal insulation, and improve membrane stability. These integrated design strategies enable the fabrication of membranes with high vapor flux, low heat loss, and strong fouling resistance, meeting the essential requirements for efficient water treatment.

#### 2.5.1. Printing and Dyeing Wastewater Treatment

With the continuous development of science and technology, the level of printing and dyeing wastewater has gradually increased. At the same time, the pollutants in the printing and dyeing wastewater are complex, such as dyes, slurries, oils, fiber impurities, and inorganic salts. At present, the commonly used membrane separation technology on the market is ultrafiltration (UF), but membrane fouling has caused obvious problems such as a decrease in the membrane rejection rate. The MD membrane can maintain 100% interception due to its excellent performance at realizing sewage separation. For example, the PVDF-HFP/PDMS composite membrane can be obtained by the electrospinning/point spraying composite process to treat wastewater composed of different dyes in DCMD [77]. The article explains the distillation of methylene blue (MB) and crystal violet (CV) performance. In long-term wastewater treatment, it has shown strong superiority.

#### 2.5.2. Desalination

With the continuous development of the world, the rapid growth of manpower, and the continuous development of industry, freshwater will become a problem we have to face. With the increase in water demand, water recovery and reuse have become ideal practices in the water supply industry. Membrane distillation is a desalination technology that can be used to recover fresh water from high-salinity streams. At present, electrospun nano-MD fiber membranes are used in simulated seawater. Researchers have achieved remarkable results and progress in the surface hydrophobicity of nanofiber membranes and the enhancement of membrane mechanical properties. Through various modifications and membrane structure optimization methods, the surface hydrophobicity of nanofiber membranes and membrane mechanical properties have been significantly improved [78,79]. Similarly, with the conventional membrane separation, the membrane distillation desalination process and AGMD are dominated by two kinds of DCMD. Shon et al. [68] reported a method of vinylidene fluoride nanofiber membrane surface modification by poly anaerobic CF_4_ plasma for AGMD. After modification, the water contact angle and wetting resistance were significantly improved.

#### 2.5.3. Heavy Metal Recycling

Traditional membrane separation technologies such as ultrafiltration (UF) and nanofiltration (NF) have been applied to treat heavy metal wastewater. But for high-concentration heavy metal wastewater, the high efficiency and low energy consumption of membrane distillation technology make MD technology the preferred treatment technology. For example, Hilal’s research group used electrospinning to prepare Al_2_O_3_/PVDF nanofiber membranes and apply them to the AGMD process to treat lead-containing wastewater. The results showed that the rejection rate of heavy metal ions reached 99.36% [80]. At the same time, the research group used double-layer electrospinning technology to prepare composite membranes to ensure their long-term stable use in high-concentration wastewater.

#### 2.5.4. ENM Diversification

Electrospinning has a high specific surface area, porosity, and permeability. The surface of the hydrophobic membrane is enhanced to a great extent, while the low flux limiting membrane distillation is effectively reduced. Membrane distillation technology has been developed for many years. At present, the electrospinning materials for membrane distillation are mainly PVDF and its polymers. More types of polymers should be electrospun into nanomaterials for molecular dynamics applications. For example, polyester with equivalent hydrophobicity has obvious advantages in terms of its low cost and low thermal conductivity [81].

#### 2.5.5. Urgent Problems

Compared to the membrane ratio produced by traditional methods, the electrospun nanofiber membrane has a high roughness and large pore size. Therefore, the degree of contamination on the membrane surface is more serious than that of traditional membranes. The study of pollutants such as inorganic salts, humic acid, and other organic matter is lacking. Also, most of the membrane distillation process takes place in the laboratory, because of the lack of membrane components necessary for the membrane distillation process; further development is needed to enhance their application in industrialization.

In membrane distillation systems, combining hydrophobic nanofiber substrates with photothermal or magnetic-responsive components can enhance water vapor flux and reduce temperature polarization. Adjustments in membrane composition, pore configuration, and surface energy contribute to differences in heat localization and vapor transport behavior under various driving modes. Both material selection and structural design play a role in determining thermal efficiency and operational performance.

## 3. Oil–Water Separation

In recent years, oil spills and the discharge of oil-bearing wastewater have caused serious damage to the ecological environment. Electrospun nanofibrous membranes have a high surface area and high porosity, and functional materials are easily added. Two-dimensional superhydrophilic/superoleophobic and superhydrophobic/superoleophilic and switchable nanofibrous membranes are introduced, and the research progress of three-dimensional separation materials (sponges and aerogels) based on nanofibrous membranes in oil–water separation is discussed.

### 3.1. Two-Dimensional Oil–Water Separation Membrane

#### 3.1.1. Superhydrophilic/Superoleophobic Membrane

A superhydrophilic/superoleophobic nanofibrous membrane is a kind of material with a superhydrophilic surface which can filter water and prevent oil from passing through. The typical superoleophobic surfaces have OCAs larger than 150° and water contact angle hysteresis (WCAH) is usually smaller than 10° [82]. In addition to the direct electrospinning of superhydrophilic/superoleophobic polymer solutions, superhydrophilic/superoleophobic nanofibrous membranes were prepared by adding hydrophilic small molecules and coating with hydrophilic polymer. In the treatment of oily wastewater, the density of water is higher, so water will prevent oil and membrane direct contact, which will slow down the flow of oil. In addition, some super pro-oil membranes are easily contaminated and blocked, resulting in a gradual decrease in separation efficiency. This has led some researchers to focus on the development of hydrophilic/oleophobic separation membranes.

**Figure 5:**
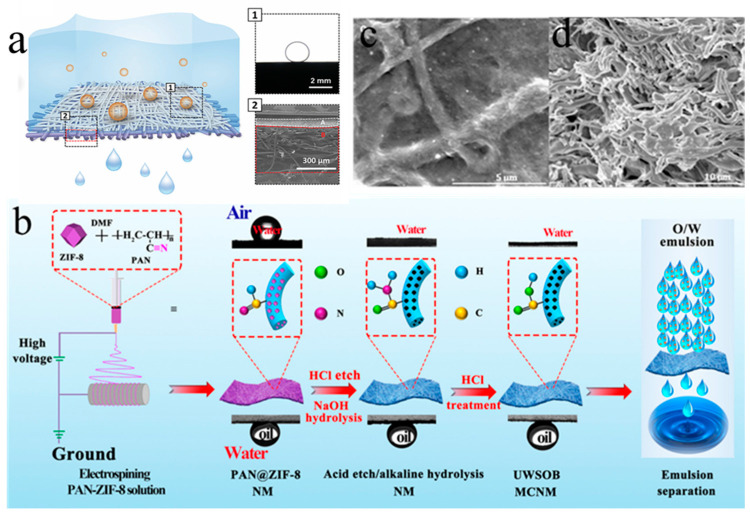
(**a**) Schematic diagram of an oil/water separation [inset 1: underwater oil contact angle (OCA); inset 2: cross-sectional scanning electron microscope (SEM) image of the NFC membrane]. Reprinted from [84] with open access license. (**b**) Fabrication process of MCNM for oil-in-water emulsion separation. Reprinted from [85] with open access license. (**c**,**d**) The SEM image of cellulose/electrospun PVDF-HFP composite surface and cross-section. Reprinted from [86] with open access license.

In recent years, many researchers have obtained superhydrophilic–superhydrophobic nanofibrous membranes by direct electrospinning polymers. The method of making nanomembrane networks is very simple and cheap, without any post-processing. Polyvinylidene fluoride and polyacrylonitrile were chosen as the materials for electrospinning, and a nanocomposite membrane for oil–water separation [83]. It was found that the blend of polyacrylonitrile and polyvinylidene fluoride had higher mechanical strength and separation efficiency. As shown in Figure 5a, Hong et al. [84] reported on the use of a nanocellulose membrane (NFC) for the continuous separation of mixtures. The cellulose membrane showed an OCA up to 150°, a high stability, and a high separation efficiency (>99%). In addition, the NFC membrane could be used repeatedly to separate oil from water under gravity drive or oil/water mixture pressure drive. The pressure-driven separation flux could reach 120,000 L·m^−2^h^−1^.

In order to obtain superhydrophilic and superhydrophobic surfaces, electrospinning technology is often combined with many polymer processing or surface/interface modification methods. As shown in Figure 5b, a novel momordica charantia nanofibrous membrane (MCNM) with superoleophobic properties is prepared by the sacrificial template method [85]. After ZIF-8 is removed, the membranes have more holes and are rougher, and the underwater super-oil-repellency is improved. The flux is up to 1580 ± 301 L·m^−2^h^−1^, and the separation efficiency of the oil-in-water emulsion is high (>99.6%). In addition, the strong mechanical and chemical stability of MCNM has great application potential in cleaning oily sewage. As shown in Figure 5c, Ejaz et al. [86] electrospun PVDF-HFP nanomembranes which were modified with ionic-liquid-regenerated cellulose. The modified nanomembrane had narrower pores and higher mechanical and chemical stability. When the content of cellulose was 15 wt%, the elastic modulus and tensile strength of the PVDF-HFP membrane were improved greatly, and the separation efficiency was up to 99.98%. The biomimetic and superwettability nanomembrane skin with lotus leaf micro/nanostructures was prepared by electrospray and electrospinning [87]. The surface of the membrane was superhydrophilic and superhydrophobic underwater due to the graded roughness and hydrophilic polymer matrix. Driven by gravity alone, it had a high flux (5152 L·m^−2^h^−1^) and a separation efficiency of over 99.93%.

#### 3.1.2. Superhydrophobic/Superoleophilic Membrane

The researchers were inspired by biological phenomena such as hydrophobic lotus leaves [88] and insect legs; they preferred to study the preparation of superhydrophobic and superoleophilic nanomembranes. A superhydrophobic surface is a hydrophobic surface with a WCA greater than 150° and a sliding angle less than 10°. There are two basic ways to prepare a superhydrophobic surface: building a rougher surface and reducing the surface free energy of the rough surface. In addition, fluorocarbon-based materials are often used to make superhydrophobic surfaces.

The material currently reported to have the lowest surface tension is fluoropolymer. Polyvinylidene fluoride, which has low surface energy, good thermal stability, and good mechanical strength, is often used as a structural hydrophobic and oleophilic membrane. Zhou et al. [89] prepared superhydrophobic and superoleophilic polyvinylidene fluoride membranes by electrospinning. The high WCA of the polyvinylidene fluoride membrane was up to 153° and the OCA approached 0°, which gave it the ability to separate emulsions efficiently. Polyvinylidene fluoride nanomembrane was also prepared by electrospinning [90]. The nanomembrane had high permeability (88166 ± 652 L·m^−2^h^−1^ bar^−1^) and high separation efficiency (>99%).

**Figure 6:**
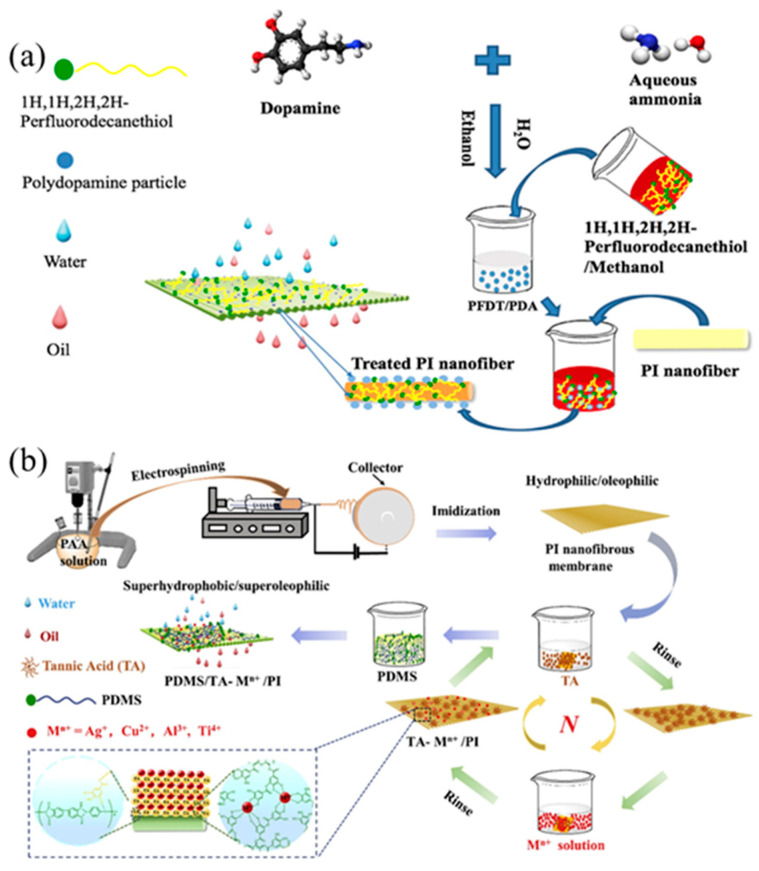
(**a**) Schematic illustration of the superhydrophobic modification of the electrospun PI nanofibrous membrane. Reprinted from [91] with open access license. (**b**) Schematic illustration shows the preparation of the PDMS/TA-Mn+/PI nanofibrous membrane, and the formation mechanism of the PDMS/TA-Mn+ coating. Reprinted from [92] with open access license.

As shown in Figure 6a, Wenjing et al. [91] obtained the superhydrophobic and superoleophilic PFDT/PDA/PI nanomembrane by immersing the polyimide nanomembrane in the mixture, and then modifying it with PFDT. The WCA of the PFDT/PDA/PI nanomembrane is greater than 153°, and the OCA approaches 0°, which gives the polyimide membrane a high flux (8018.5 ± 100 L m^−2^ h^−1^) and separation efficiency (>99%) in oil–water separation. As shown in Figure 6b, a polyimide electrospun nanomembrane is prepared, which is immersed in an aqueous solution of tannic acid (TA) and metal ions, and then coated with PDMS to form a hydrophobic nanomembrane [92]. The tannic acid Al^3+^-based superhydrophobic membrane gives the membrane the properties of impact resistance, ultraviolet shielding, and self-cleaning. After 20 separation cycles, the flux of 6935 L·m^−2^h^−1^ is still high and the separation efficiency is over 99%. Su et al. [93] prepared superhydrophobic nanomembranes by simultaneous electrospray silicon dioxide colloidal and electrospinning polyvinylidene fluoride solutions. The membrane had a WCA of 163° and the angle of slip was only 3°, which had potential to be used in applications. Qing et al. [94] prepared a polytetrafluoroethylene nanomembrane by electrospinning. After sintering, the polyvinyl alcohol polymer was decomposed to form a polytetrafluoroethylene nanomembrane. The WCA of the polytetrafluoroethylene membrane was 155.0° and the low sliding angle was 5.1°. Excellent mechanical strength, a high permeation flux of 1215 L·m^−2^h^−1^, and high corrosion resistance and stability make polytetrafluoroethylene membranes promising for industrial oil–water separation.

The selective wettability and separation efficiency of superhydrophobic/superoleophilic membranes are highly dependent on their surface topography and chemical composition. The presence of micro-/nano-hierarchical structures and low-surface-energy functional groups promotes directional oil penetration while repelling water droplets. Furthermore, the interconnected fibrous network facilitates rapid oil transport and drainage, contributing to high separation rates under gravity-driven conditions. These structural features also help to maintain the long-term stability and recyclability of the membranes in oil–water separation applications.

#### 3.1.3. Switchable Oil–Water Separation Membrane

To meet the different separation requirements in industrial production, it is expected that a separation membrane should be prepared which will be low-cost and high-utilization, and which will be switchable. Electrospinning can build smart membranes with extreme wettability, which are often used to prepare switchable oil–water separators.

The preparation of a superoleophobic under water and superhydrophobic under oil nanomembrane is of great significance for the separation of mixtures. By prewetting in air with water and oil, respectively, the membranes show superhydrophobicity under water or superhydrophobicity under oil. Weimin et al. [95] used waste cigarette filter tip as the material to prepare a cigarette filter tip coating net by a simple electrospinning method. After prewetting with water or oil, the prepared carboxymethyl cellulose can achieve the special superhydrophobic wetting properties under water or under oil without further chemical modification. As a result, an exclusive oil/water mixture and emulsion are separated by a coating screen on the filter tip of a cigarette. Also, the efficiency of CFCMs for immiscible oil/water mixtures and emulsions is still higher than 99.9% after several cycles of testing. The work not only provides a method for separating oil from water, but also reduces the pollution of waste cigarette filters to the environment, and is more energy-saving and provides environmental protection. Zhang et al. [96] synthesized a COF-DhaTab/polyacrylonitrile nanocomposite membrane with biological non-linear multi-node structure by a simple blend electrospinning method. Liao et al. [97] prepared a PVDF-SiO_2_ nanomembrane by electrospinning. The membrane was super-amphiphilic in air and superhydrophobic under water and under oil, meaning it could handle all kinds of oil–water blends at ultra-low pressure. The flux of the membrane was 2000 L·m^−2^h^−1^, the separation efficiency was 99.99%, and the stability was excellent under harsh conditions.

**Figure 7:**
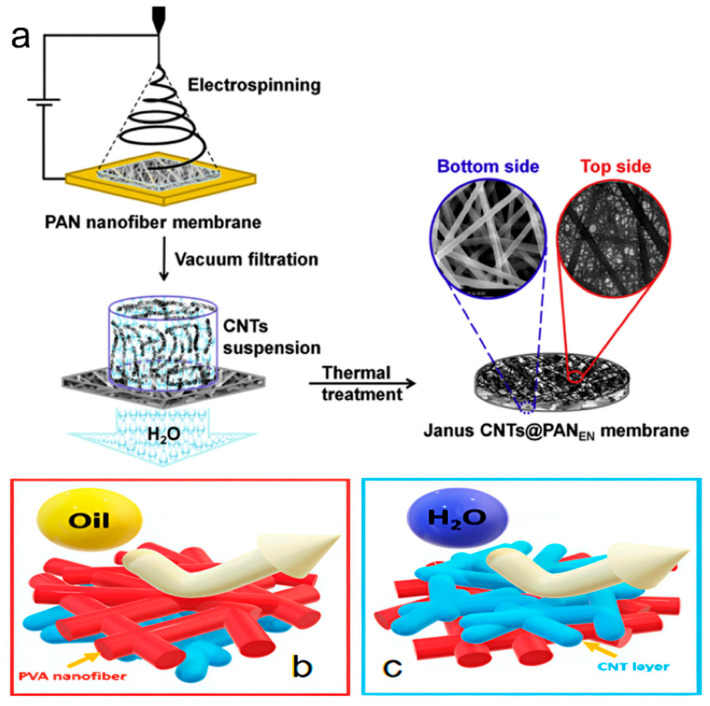
(**a**) Schematic illustration of the fabrication process for Janus CNTs@PANEN membranes. Reprinted from [98] with open access license. (**b**,**c**) FTIR spectra with PFOTS concentration variation. Reprinted from [99] with open access license.

A Janus membrane is a kind of membrane material with different properties on both surfaces and asymmetric wetting characteristics on both surfaces. Janus membranes are often constructed by electrospinning, with asymmetric wettability on both sides. The double-layer membranes can be used to separate oil-in-water or oil-in-water emulsions. The method of preparing a Janus membrane by electrospinning is to coat the hydrophilic/hydrophobic layer on the surface and graft hydrophilic/hydrophobic polymer on the surface. A Janus membrane is composed of a hydrophilic polyacrylonitrile (PAN) electrospun nanomembrane and a one-sided hydrophobic carbon nanotube network coating (CNTS) [98] (Figure 7a). A small number of carbon nanotubes enables the membrane to exhibit good stability. Due to the hydrophobic properties of the PAN membrane and the lipophilicity of carbon nanotubes, the carbon nanotubes@polyaniline membrane has switchable oil/water separation performance. The hydrophilic membrane is prepared by electrospinning of polyisopropylacrylamide and polyvinylidene fluoride, and then electrospinning of hydrophobic polyvinylidene fluoride on the hydrophilic membrane to form the Janus membrane [99]. The structure of the Janus membrane can not only improve the flux but also can also realize switchable separation. When the polyvinylidene fluoride is facing up, it separates the oil and water in the oil emulsion. When the polyisopropylacrylamide polyvinylidene fluoride faces up, water and oil can be separated in an aqueous emulsion. Pornea et al. [100] applied functionalized carbon nanotube (F-CNT) layers to a hydrophilic polyvinyl alcohol (C-PVA) membrane through a simple vacuum filter. The Per-fluorooctyl triethoxysilane on carbon nanotubes (CNTSs) resulted in a superhydrophobic interface. Thus, oil-in-water emulsions and water-in-oil emulsions can be separated by changing the direction of the interface.

The interfacial modification may affect the inherent function of the membrane, and the surface coating is easily removed by a solvent or mechanically [101]. Therefore, it is a very good choice to embed the membrane by electrospinning. A Janus membrane is made by electrospinning a polylactide/carbon nanotube membrane and electrospinning a polylactide/silica nanofluid membrane on one side. The wettability of the Janus membrane is completely the opposite, the membrane is hydrophobic (water contact angle > 140°), and the silicon dioxide membranes with organic anions and cations are (WCA ≈ 0°). The asymmetric Janus membrane obtains a high throughput (1142–1485 L·m^−2^h^−1^) and high separation efficiency (>99%).

Because the wettability of the membrane surface can be changed by the change in the material environment, more and more stimulus-response membranes have been widely used in oil–water separation. Smart surfaces have been used to separate oil from water by changing external stimuli such as pH, light, temperature, and gases. Electrospinning technology with high porosity, low cost, and easy operation is often used to construct polymer surfaces with special structures.

Due to the different pH of water, pH-responsive nanomembranes have attracted the attention of researchers. The copolymer polymethyl acrylate-block-Poly (4-vinylpyridine) is commonly used as a pH-responsive smart membrane. Li et al. [102] prepared PMMA-b-P4VP/SiO_2_ nanomembrane mats sensitive to pH by electrospinning. The pH response of underwater hydrophilic PMMA combined with P4VP endows the membrane with swappable surface wettability. The silicon dioxide is filled to improve the stability, mechanical strength, and permeability of the composite membrane. Initially, only gravity-driven oil can pass through the membrane, and reverse separation can be achieved by wetting the membrane with water of a pH equal to 3. The PMMA-b-P4VP membrane has potential in practical applications due to its high separation efficiency, excellent recyclability, and antifouling performance. Another key to improve the separation efficiency is to develop swellable wettability membranes with high permeability flux. The switchable wettability polystyrene membrane was prepared by electrospinning and UV photografting. Dmaea and Tfoa were selected as grafting monomers, and a low proportion of fluorinated acrylic polymer segments were designed [95]. The synergistic action of the pH-sensitive polymethyl acrylate and the hydrophobic polytetrafluoroethylene enabled the wetting properties to be reversely switched depending on the pH change. The membrane could effectively separate the mixture; the flux of oil was 10,186.8 L·m^−2^h^−1^, and the gravity-driven separation efficiency was 99.2%. By changing wettability from lipophilicity to hydrophilicity especially, excellent separation performance for other oil–water mixtures with high viscosity has been achieved.

**Figure 8:**
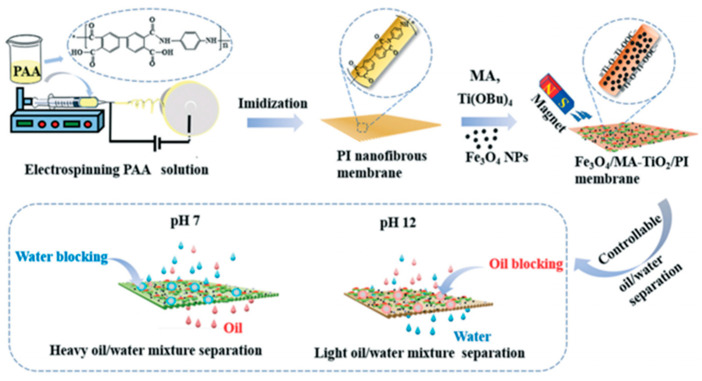
Preparation and application of Fe_3_O_4_/MA-TiO_2_/PI membranes at different pH values. Reprinted from [91] with open access license.

In addition, there are many reports on the use of pH as a switchable oil–water separator switch [103]. As shown in Figure 8, a flexible and pH-sensitive magnetic Fe_3_O_4_ matitanium dioxide polyimide electrospun nanomembrane (ENM) [91] was prepared by electrospinning and green dip coating. The magnetic pH-responsive intelligent separation membrane was prepared by coating a mixture of myristic acid (MA) and titanium dioxide and pregel solution of Fe_3_O_4_ nanoparticles on the substrate of polyimide with excellent flexibility, mechanical strength, and thermal stability. Due to its rough classification structure and wettability, the hybrid membrane could be used to separate heavy/light oil from water by adjusting the pH of the water medium. A pH-responsive smart dendrimer nanomembrane [104] was prepared by homogeneous solution polymerization and electrospinning. The dendrimer nanomembrane increased the effective contact area between the solution and membrane and improved the pH response sensitivity of the nanomembrane. The surface oil/water wettability of the membrane could be changed by changing the pH value of the water medium. More importantly, both separations exhibited excellent efficiency and fluxes under the action of gravity alone.

Photo-responsive membranes have great potential in water purification, wastewater treatment, and oil–water separation by integrating membrane filtration and photocatalysis. The titanium dioxide has a relatively high bandgap energy (3.2 ev), good chemical stability, and low cost. In recent years, titanium-dioxide-based catalysts have received considerable attention for the development of UV-responsive photo-responsive membranes. Shami et al. [105] have proposed a one-step process to prepare light (UV/sun)-induced polyvinylidene fluoride silicon dioxide textiles by electrospinning. Induced by UV/sunlight, the wettability of the membrane changes rapidly, and reverses between superhydrophobicity and superhydrophilicty. Sundaran et al. [106] reported a reduced graphene oxide–titanium dioxide–polyurethane (rgo-TiO_2_-PU) nanomembrane, which has visible light response and anti-pollution properties. The optimized membrane has a water flux of 12,810 ± 49.65 L m^−2^h^−1^, an oil cut rate of 85.50%, and a flux recovery of 88.10%. The light-responsive nanomembrane has excellent antifouling properties, and the organic molecules can be removed by titanium dioxide photocatalysis.

Researchers have also studied the temperature-responsive intelligent switchable nanomembrane, which can realize oil–water separation under controlled conditions. In general, temperature-responsive membranes are prepared by grafting or copolymerizing thermosensitive polymers (or groups) onto molecular chains. PNIPAAM, as a widely studied thermal-responsive polymer, is often used to prepare temperature-switched wettability nanomembranes. Liu et al. [107] prepared polycaprolactone membranes by electrospinning, and modified the surface of the membranes by surface-initiated atom transfer radical polymerization (ATRP)-grafted polyisopropylacrylamide (PNIPAM) brushes. The regenerated cellulose nanomembrane was prepared by electrospinning; subsequently, a thermo-responsive PNIPAAM was grafted onto the surface of a nanomembrane to form a polymer chain/brush by the surface-initiated atom transfer radical polymerization method [108]. In response to different temperatures, the PNIPAAM-grafted nanomembrane exhibited switchable superhydrophilic/superhydrophobic properties at the water–oil–solid interface and excellent controllable oil–water separation performance.

A temperature-sensitive copolymer poly (methyl methacrylate)-block-polyisopropylacrylamide (PMMA-b-PNIPAAM) nanomembrane was prepared by electrospinning [109]. The thermo-sensitive component PNIPAAM gave the membrane oil/water wettability with adjustable temperature. The porous structure played an important role in the wettability of the electrospun membrane: the surface roughness of the membrane was increased by the randomly entangled membranes, and the wettability and anti-wettability of the membrane were further improved by the high-density micropores. By adjusting the temperature, the prepared membrane could realize the oil–water separation of gravity drive with an efficiency higher than 98%, achieving higher fluxes of about 9400 L h^−1^m^−2^ for water and about 4200 L h^−1^ m^−2^ for oil.

In recent years, gas-responsive membranes have attracted much attention. The CO_2_ conversion membrane is attractive because of its non-toxicity, lack of pollution, and low cost. Abbas et al. [110] prepared CO_2_-shs synthetic membranes made from poly (methyl methacrylate) copolymer. Instead of argon recovery, they used CO_2_ as the hydrophilic trigger and electric potential as the hydrophobic trigger. Their research proves that the effect of using electric potential as the trigger of a hydrophobic state is a better option. The wettability of SHS is reversible, which indicates that SHS can switch between hydrophilicity or hydrophobicity in alternating bubbling CO_2_ and EP. Che et al. [111] prepared poly (methyl methacrylate) copolymer and prepared polymer nanomembranes (SNMs) by electrospinning. The presence of the membrane network in the electrospinning process increased the roughness of the membrane, and the micropores on the surface allowed the gas to pass through. Under the alternating stimulation of CO_2_/N_2_, the membrane could be adjusted between hydrophobicity/hydrophilicity, and the selective wetting behavior induced by CO_2_ could be realized successfully. Therefore, the use of carbon dioxide as a driving force in oil–water separation has great prospects.

### 3.2. The 3D Porous Composites Were Prepared on the Substrate of the Electrospinning Membrane

Two-dimensional (2D) membranes primarily exhibit a thin-layered planar structure, where their pores and performances are macroscopically constrained by their two-dimensional nature. Although all membrane structures possess certain three-dimensional characteristics, the term “3D” in 3D porous composite materials refers to the complexity of their spatial architecture and the multi-dimensional interconnectivity of their pore networks. These pore networks extend not only in the plane but also feature rich channels and interconnected structures in the vertical direction. Such a three-dimensional porous network endows the material with a higher specific surface area, enhanced permeability, and more complex performance characteristics, offering significant advantages in applications such as filtration, mass transport, and mechanical properties. During the long-term operation of the 2D oil–water separation membrane, the flux and separation efficiency will be reduced due to the pollution of surfactant adsorption and pore plugging. Three-dimensional porous materials (aerogels, sponges, etc.) are effective candidates for oil–water separation due to their high porosity, high curvature, and appropriate pore size.

#### 3.2.1. Three-Dimensional Porous Sponges

Lv et al. [112] used PMF sponges with high porosity, ruggedness, and thermal stability as the substrate to insert silicon dioxide membranes into the sponges immersed in a homogenized membrane dispersion to modify 3Si-PMF. Lactate de-hydrogenase nanoparticles make sponges superhydrophilic, and silicon dioxide membranes regulate pore size by overlapping with PMF hosts. The pore size of the sponge decreases, but the porosity is still above 97.8%. The resulting modified sponge can effectively separate various oil/water mixtures, including surfactant emulsions, only under the action of gravity. The porous sponge has high permeability flux and an excellent oil discharge rate (>99.46%). In a word, the porous sponge has a good stability, antifouling, and recycling rate, and is expected to be a candidate material for oil–water separation in practical application. Hao-yang et al. [113] prepared 3D-HOMs from silicon dioxide sponge by self-assembly electrospinning. Thanks to continuous silicon dioxide membranes and graded cobalt microstructures, the modified silicon dioxide sponge (MSS) has ultra-hydrophobic and oil-selective properties, low packing density (49 mg cm^3^), a big surface area, high absorptivity, ultra-high heat resistance, and remote controllability through magnetic field manipulation. By bringing the internal structure to the contact interface, the separation efficiency is improved by compressing the multi-pipe system. Therefore, the MSS has the potential for use in oil–water separation.

#### 3.2.2. Three-Dimensional Porous Aerogels

**Figure 9:**
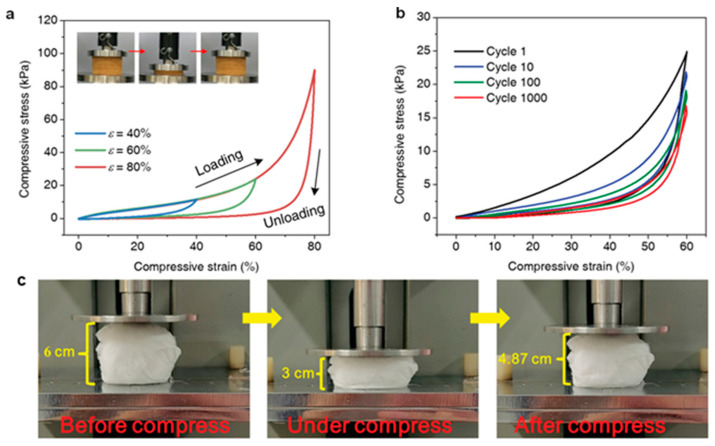
(**a**,**b**) Multicycle compression test results of membrane aerogels. Reprinted from [114] with open access license. (**c**) Schematic illustration of the compression strength. Reprinted from [116] with open access license.

By combining electrospun nanomembranes and freeze-forming techniques, fibro-isotropy-combined elastic restructuring aerogels were produced [114]. Polyacrylonitrile and silicon dioxide were selected as substrates, homogenized in silicon dioxide at 70 °C to form a homogeneously dispersed nanofabrication, and finally, aerogel was formed by freeze-drying. To make the aerogel stronger, the unbonded aerogel was heated at 240 °C, which made the membrane aerogel elastic. The aerogel had an ultra-low density (<30 mg cm^−3^), could recover from 80% compressive strain (Figure 9a,b), exhibited superhydrophobic and superolephilic wettability, and had high porosity curvature. Under the action of gravity only, aerogels had high throughput (maximum 8140 ± 220 L m^−2^ h^−1^) and high separation efficiency.

#### 3.2.3. Three-Dimensional Porous Membrane Scaffolds

Hem et al. [115] used a simple hydrothermal method to prepare 3D foaming polyacrylonitrile (PAN) membrane scaffolds by adding nanosized zinc oxide (ZnO) and conducting surface modification with dodecyl triethoxysilane (DTEOS). The hydrophobic PAN/ZnO membrane was prepared by modification of three-dimensional PAN/ZnO membrane with (DTEOS). Compared to the 2D PAN/ZnO membrane, the modified 3D hydrophobic PAN/ZnO composite scaffolds had excellent oil absorption properties, and the oil attached to the 3D membrane scaffolds could be easily expelled by simple mechanical extrusion. The study shows that the hydrophobic and olephilic three-dimensional PAN/ZnO membrane scaffold is the best choice for oil spill treatment because of its high oil absorption performance, excellent oil–water selectivity, and high buoyancy. Di et al. [116] prepared a 3D solid poly (l-lactide) (PLLA) membrane material driven by sodium dodecyl benzene sulfonate (SDBS) through electrospinning and evaporative welding, which was used for oil–water separation. During electrospinning, SDBS enhances the electrical conductivity of PLLA membranes to form a three-dimensional structure. Three-Dimensional porous membrane scaffolds have high porosity (99.91%), high compression strength (Figure 9c), and excellent oil–water separation efficiency. These results indicate that 3D PLLA-SDBS membrane scaffolds have potential applications in oil–water separation.

In oil–water separation, tailoring surface wettability through chemical modification or hierarchical roughness is effective for achieving selective permeation. Membrane asymmetry, interfacial energy gradients, and fiber alignment can influence the direction and efficiency of phase transport. Variation in structural features such as pore size and surface texture offers multiple pathways for controlling liquid separation behavior under different external forces.

## 4. Solar Evaporation

Since interfacial heating was proposed in recent years, solar-driven water evaporation has been considered a promising technology for seawater desalination. This technology accelerates the evaporation of water by converting absorbed sunlight into heat, uses photothermal materials to concentrate heat at the water–air interface, and reduces the flow of heat to the lower layer of moisture, thereby reducing costs while protecting the environment. However, for the method using sunlight and heat to evaporate water, the prerequisite is to have convenient and effective light-to-heat conversion materials. Electrospinning technology is usually used to prepare a flexible nonwoven film with an entangled scaffold structure composed of entangled microfibers and nanofibers. By changing the composition and spinning conditions of the spinning dope, the different properties of the electrospun nanofiber membrane can be controlled relatively easily. Based on these principles, electrospinning will become an important technology for preparing high-efficiency solar energy conversion materials. The research progress for two-dimensional and three-dimensional electrospun nanofiber membranes in solar evaporation is introduced.

### 4.1. Two-Dimensional Solar Thermal Conversion Material

As the pivotal component in solar-powered seawater desalination, photothermal materials have undergone a systematic evolution from photothermal nanoparticles to two-dimensional (2D) photothermal materials, and subsequently to 2D photothermal membrane architectures. In recent years, 2D photothermal composite materials have garnered increasing attention owing to their advantages of low cost, mechanical flexibility, scalability, and portability.

#### 4.1.1. Materials Prepared by Doping Method

Ding et al. [117] synthesized an innovative double-layer polylactic acid (PLA) fiber membrane double-layer interface evaporation module loaded with Chinese ink nanoparticles. An electrospun layer of micro-scale-diameter fibers with enhanced hydrophilicity was used as the water transport layer, and the solution of the electrospun layer of nano-scale-diameter fibers loaded with Chinese ink nanoparticles was used as the evaporation interface. According to the test, under light intensity, the evaporation rate of the double-layer membrane with 2 wt% Chinese ink nanoparticles was 1.29 kg cm^−2^h^−1^, and the efficiency was 81.0%. Tong et al. [118] introduced the incorporation of Au nanocages (AuNCs) into polyvinylidene fluoride (PVDF) electrospun nanofibers. Because PVDF is highly hydrophobic, its nonwoven mat will naturally float on the water surface. When irradiated with a near-infrared (NIR) laser of 808 nm, the water on the surface is heated to evaporate, while most of it remains at an ambient temperature. Under natural solar radiation, the water evaporation efficiency can reach 67.0% and 79.8%.

Chala et al. [119] introduced a melt-electrospun-reduced tungsten oxide/polylactic acid (WO2.72/PLA) fiber membrane with improved near-infrared (NIR) photothermal conversion performance. The WO2.72 powder nanoparticles were incorporated into the PLA matrix by melt processing, and then the composite material was extruded into wires using a single screw extruder. Subsequently, fiber membranes were prepared from WO2.72/PLA-composite-extruded filaments by melt electrospinning. This technology is economical and environmentally friendly. Under a single period of sunlight, the water evaporation efficiency reached 81.39%, which is higher than the evaporation efficiency of pure PLA fiber membrane and bulk water.

#### 4.1.2. Material Prepared by Carbonization Method

At present, a variety of materials have been developed in the field of photothermal conversion, such as nanocomposite phase change materials [120], conductive polymer materials [121], and carbon-based materials [122]. Although these types of materials have achieved high photothermal conversion efficiency, the cost of mass production of plasmon or semiconductor materials is relatively high, which reduces the scope of their application. Compared with them, carbon-based materials have high light absorption, low cost, and good chemical stability (resistance to acid, alkaline, or salt solutions), and flexible manufacturing processes (electrospinning methods) that enable them to achieve cost-effective solar-driven evaporation.

Guo et al. [123] introduced the development of graphene-oxide-functionalized polyvinyl alcohol EFM (GO/PVA EFM) as a light-to-heat conversion material through hybrid electrospinning technology. GO/PVA EFM was composed of uniform nanofibers, in which GO was fixed in the fiber as a light-absorbing component. Under a single period of sunlight, the evaporation rate of GO/PVA EFM with a GO concentration of 5% in purified water can reach 1.40 kg cm^−2^h^−1^, and the energy conversion efficiency can reach 90.0%. For simulated seawater, the optimal evaporation rate can reach 1.42 kg cm^−2^h^−1^, and the corresponding energy conversion efficiency is up to 94.2%.

Peng et al. [124] introduce a membrane prepared by the electrospinning method that has a porous nanofiber structure, and rGO has a high solar light absorption rate; the two are combined to prepare a high-performance electrospun membrane based on rGO. They select polyacrylonitrile particles (PANs) as the raw materials, then the GO/PAN nanofiber membrane is chemically reduced to rGO/PAN membrane in L(+)-ascorbic acid (Vc) solution [125]. The light absorption rate can reach 80%. When placed on a heat insulation board made of polystyrene foam, it can convert 89.4% of light into heat, which is significantly higher than the pure PAN membrane and GO/PAN membrane.

**Figure 10:**
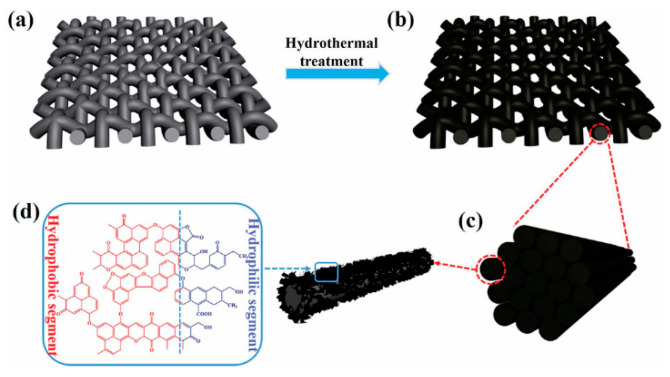
The schematic diagram of the carbon fiber hydrothermal carbonization mechanism. (**a**) The original carbon fibers, (**b**,**c**) carbon fibers modified by hydrothermal treatment, (**d**) the insoluble inter-bonded aromatic amphiphilic macromolecules derived from glucose. Reprinted from [130] with open access license.

Gao et al. [126] introduced an evaporator composed of electrospun hydrophobic polyvinylidene fluoride (PVDF) nanofibers and hydrophilic carbon black/polyacrylonitrile (CB/PAN) composite nanofiber layers, which is low cost, can float, and is durable. It has a solar energy conversion efficiency of 82.0% under one period of sunlight. Wu et al. [127] introduced a method of selecting polyacrylonitrile particles (PANs) as raw materials and using electrospinning to prepare continuous and ultrafine porous membranes. Among them, the continuous long fibers in the porous carbon membrane can transport water over long distances, so real-time and sufficient water supply can be achieved [128]. Its light absorption efficiency is very high, in the wavelength range of 250 to 2500 nm, which can reach 95%. The water evaporation rate of the carbonaceous membrane can reach 1.33 kg cm^−2^h^−1^ under normal conditions, and the solar evaporation efficiency can reach 81.71%, which can meet the general conditions of use; fresh water can also be recovered in acidic and alkaline solutions. The ultra-sturdy solar steam system with carbon fiber modified by hydrothermal carbonization can efficiently purify dyed organic solvents, oil-in-water emulsions, and seawater. Due to the introduction of a layered carbon coating, the modified carbon fiber shows excellent mechanical strength, good wettability [129], and excellent light absorption, so that CFs can pump water and organic solvents and other media through capillary action (Figure 10) [130]. The evaporation rate of simulated seawater is as high as 1.47 kg m^−2^h^−1^; steam generation efficiency can reach 92.5%. In addition, the polar carbonized coating provides excellent water transfer capacity, and it can avoid salt precipitation and scaling by operating overnight.

The photothermal performance of electrospun membranes in solar-driven evaporation is closely related to both the intrinsic optical properties of the incorporated materials and the membrane’s structural design. Materials such as carbon nanotubes and reduced graphene oxide provide high broadband light absorption and photothermal conversion, while the porous and rough fiber network promotes localized heating, water pumping, and vapor escape. The multi-layered architecture also contributes to thermal confinement and efficient vapor release. These structural features collectively enhance the evaporation rate and energy utilization under solar irradiation.

### 4.2. Three-Dimensional Solar Thermal Conversion Material

The evaporation system usually adopts a double-layer structure: the top photothermal layer is used for light-to-heat conversion, and the bottom support layer is used to reduce heat loss [131]. Although this structure has made significant progress in improving evaporation efficiency, it usually uses 2D materials (flat membrane, paper, thin membrane), and the evaporation rate and solar heat conversion efficiency of these materials have almost reached the upper limit [132].

#### 4.2.1. Three-Dimensional Porous Carbon-Based Materials

Layered graphene and polymethylmethacrylate were used to fabricate a composited membrane (GF) by electrospinning, which acted as a solar absorber. Together with a water transporter (polyurethane sponge) and a thermal insulator (polystyrene foam), the GF-based evaporator was constructed for solar evaporation. Taking advantage of the porous three-dimensional structure of GF, the light path could be extended, rendering the broadband solar absorption efficient (92%) [133].

**Figure 11:**
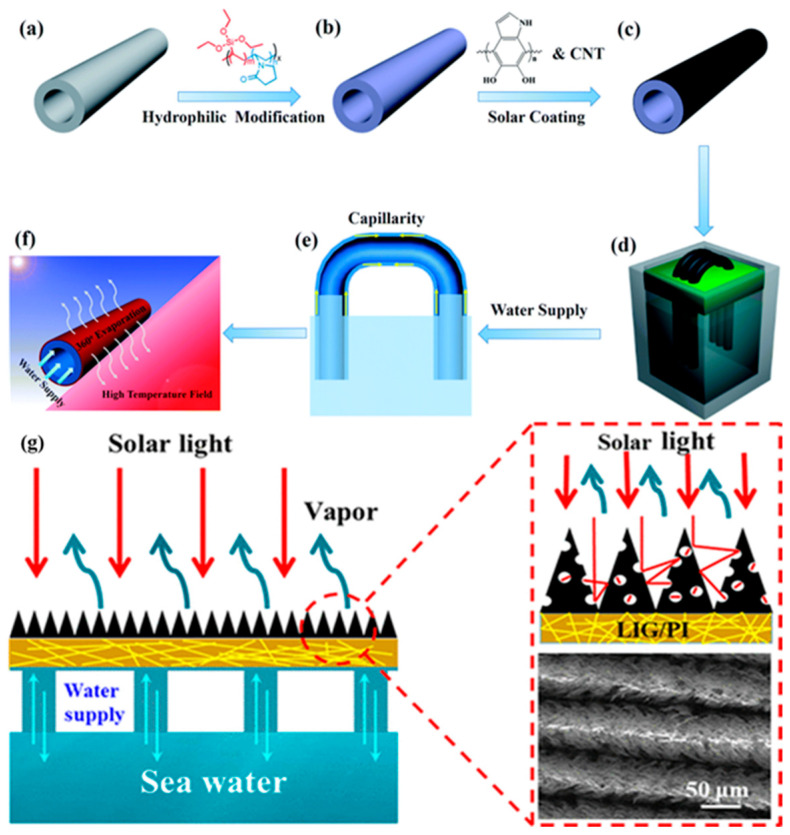
Fabrication process of arch-like PTFE/CNT membrane evaporator. (**a**) Hydrophilic modification via interface cross-linking of PVP-VTES; (**b**) CNT coating on PTFE hollow fibers via PDA mediation for enhanced solar–thermal conversion; (**c**,**d**) PTFE/CNT hollow fibers arrayed to form the arch-like evaporator; (**e**) water self-wicking via the capillary force along the directional nanofibrous structure; (**f**) a full 360-degree evaporation driven by incident solar energy plus extra heat from the support. Reprinted from [138] with open access license. (**g**) Schematic diagram of porous laser-induced graphene (LIG)/polyimide (PI) photothermal membrane photothermal water evaporation. Reprinted from [134] with open access license.

A porous laser-induced graphene (LIG)/polyimide (PI) photothermal membrane with a three-dimensional (3D) structure is prepared by electrospinning technology and laser ablation technology [134]. The overall 3D structure increases the evaporation area and reduces the energy loss caused by the diffuse reflection of light (Figure 11g). For example, cup-shaped [135] and conical-shaped [136] 3D structures can make light reflect multiple times, thereby helping to improve light absorption [137]. The LIG/PI membrane is supported by a cotton core, which has a strong capillary effect and can transport water. The water evaporation rate reaches about 1.42 kg m^−2^h^−1^, while the solar heat conversion efficiency reaches about 92.55%.

An arched evaporation system is composed of a generally shaped array of polytetrafluoroethylene (PTFE) hollow fibers modified by carbon nanotubes (CNTs) [138]. The PTFE hollow fibers are hydrophilically modified by PVP-VTES to form a capillary water pipe network. Then polydopamine (PDA) is encapsulated with carbon nanotubes (CNTs) (Figure 11a–f). PTFE hollow fiber has good flexibility and durability, and can be bent to a great extent. Hollow fibers can directly absorb energy from sunlight. Under natural conditions, experimental tests show that the fiber evaporation rate of 15 stair arrays based on carbon cloth is as high as 2.15 kg m^−2^h^−1^; the evaporation system can produce 10~16 kg m^−2^ of fresh water per day.

#### 4.2.2. Three-Dimensional Porous Aerogel

By combining the electrospinning technology and the fiber-freezing molding technology, an elastic ceramic-based nanofiber aerogel with a porous structure is produced. The technology is composed of a vertically arranged container and a porous container wall. Under the action of convection and diffusion promoted by this unique cell structure, the aerogel exhibits excellent salt resistance even in 20% salt water and under six periods of sunlight, and there are no salt crystals on the surface of the aerogel. The advantage is that due to the synergistic effect of the promising structure and the absorbance of carbon nanotubes, the aerogel has a high absorbance of up to 98% and excellent evaporation performance [139].

In solar-driven evaporation, membrane systems that combine porous support layers with light-absorbing coatings can promote efficient interfacial heating and water vapor generation. The spatial arrangement of photothermal materials, along with membrane thickness and pore architecture, influences energy transfer and evaporation rate. Structural confinement and surface topology are key factors in regulating thermal management at the evaporation interface.

## 5. Pollutant Removal

Membrane technology is a new water treatment technology. Among various membrane preparation technologies, electrospun nanomembranes have the advantages of small pores, high porosity, and large specific surface area, so many polymer nanomembranes have been prepared by electrospinning technology. Therefore, electrospun nanomembranes have been widely studied in removing harmful substances from wastewater. Moreover, their environmental and economic impacts should not be overlooked. The use of volatile organic solvents and prolonged operation times may lead to increased energy consumption, environmental pollution, and higher production costs, especially in large-scale applications. Incorporating sustainability considerations and environmental pollution factors such as catalytic degradation of pollutants, process efficiency, and material recyclability can significantly enhance the overall assessment of electrospun membranes. Malara et al. [140] evaluate the environmental footprint of electrospun polymeric nanofiber production using simplified LCA. They show that key process parameters including solvent volatility, solution concentration, and spinning duration significantly affect global warming potential and ecotoxicity. For instance, higher solvent volatility and prolonged electrospinning markedly increase the environmental impact. The study recommends adopting low-VOC solvents, optimizing solution viscosity, and shortening fiber-processing time to reduce ecological burden. In this paper, the application progress of nanomembranes in the photocatalytic degradation, adsorption, and filtration of pollutants is reviewed, and the more advanced treatment of pollutants has been shown to have prospects.

### 5.1. Photocatalytic Process

Dyes are colored substances. A dye must be able to make a certain color adhere to the membrane, and it must be difficult for color to fall off and change. However, dyes may be allergenic, carcinogenic, toxic, contain heavy metals exceeding the standard, contain formaldehyde exceeding the standard, etc. When discharged directly into water, the water quality will be seriously poor and people’s health will be affected. Among dyes, a great challenge is degrading them into harmless substances. Therefore, it is very important to remove dyes from wastewater to protect living things and the environment. Photocatalysis is based on the redox ability of a photocatalyst under ultraviolet irradiation to purify pollutants.

**Figure 12:**
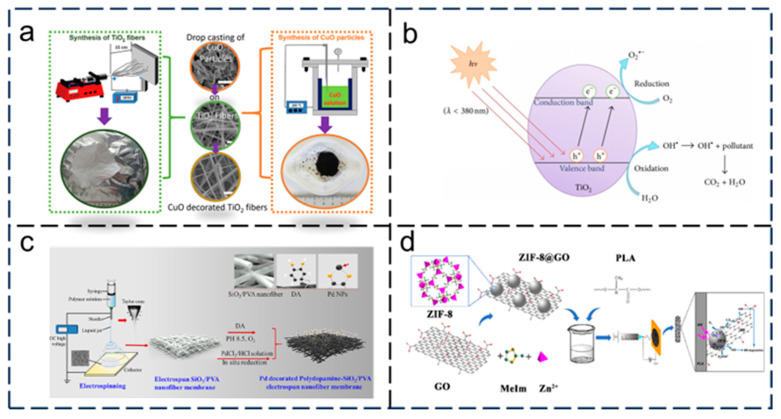
(**a**) Schematic illustration of the synthesis procedure for CuO particle–TiO_2_ membrane heterostructured photocatalysts. Reprinted from [145] with open access license. (**b**) General mechanism of the photocatalysis. Reprinted from [144] with open access license. (**c**) Schematic preparation of Pd-decorated polydopamine-SiO_2_/PVA electrospun nanomembrane via pinning, DP deposition, and an in situ reduction process. Reprinted from [146] with open access license. (**d**) Poly (lactic acid) (PLA) electrospun membranes immobilized with zeolitic imidazole framework/graphene oxide hybrid (ZIF-8@GO) are fabricated via electrospinning. Reprinted from [147] with open access license.

Common catalysts are metal oxides and sulfides. Among them, TiO_2_ has the best and most comprehensive properties and is widely used. Ji et al. [141] introduced the advantages of titanium dioxide as a catalyst, and the TiO_2_/ACF composite material is used because TiO_2_ semiconductors can produce hydroxyl radicals OH^+^ and O^−2^ with high activity and strong oxidation in aqueous solution, and organic pollutants can be adsorbed on the surface of TiO_2_, oxidized, and degraded. Miren et al. [142] prepared TiO_2_/PA6 nanomembrane by electrospinning, and found that it has excellent photocatalytic performance under ultraviolet irradiation. Wei Li et al. [143] prepared a H_4_SiW_12_O40(SiW_12_)/cellulose acetate composite nanomembrane by electrospinning with cellulose acetate as a carrier. Under ultraviolet irradiation, the prepared composite membrane showed higher photocatalytic activity for the decomposition of tetracycline and methyl orange than pure SiW_12_. Ting Feng et al. [144] summarized the photocatalytic mechanism of one-dimensional (1D) titanium dioxide nanostructures (Figure 12b) and the photocatalytic activity of photocatalysts can be further enhanced by modification with metal ions, metal oxides, or inorganic elements.

There is also some photocatalysis which occurs by surface modification of membranes and synthesis of heterojunctions. Büra et al. [145] synthesized CuO plate particles/titanium dioxide membrane heterostructures by the hydrothermal method/electrospinning method (Figure 12a), and found that the degradation behavior was affected by the number of copper oxide particles in the sample. Yin et al. [146] studied the novel palladium-modified polydopamine-TiO_2_/PVA electrospun nanomembrane (Figure 12c), and with the deposition of dopamine, palladium nanoparticles were reduced in the green environment, thus avoiding water pollution again. Palladium nanoparticles reduced in situ are uniformly distributed on the nanomembrane, thus avoiding aggregation and improving catalytic activity. Dai et al. [147] found that a polylactic acid/ZIF-8@GO electrospun membrane (Figure 12d) has a higher tensile strength than pure polylactic acid, and the composite membrane also shows good removal ability through adsorption and photocatalytic degradation. Lou et al. [148] described the photodegradation of polyvinylidene fluoride (PVDF)/titanium dioxide (TiO_2_) nanomembranes under visible light, proving that TiO_2_-based nanomembranes have catalytic ability under low-energy visible light. Recently, Zhao et al. [149] combined a water-based hydrophobic material and a photosensitive inorganic nanoparticle coating with an electrospun nanomembrane matrix to prepare a waterproof breathable membrane with high performance and strong photocatalytic degradation ability.

### 5.2. Adsorption

Adsorption refers to an interface phenomenon in which the concentration of one or more components in the bulk phase (including gas, liquid, and solution) is different from that at the interface. When the concentration of a component on the interface layer is greater than its concentration in the bulk phase, it is called positive adsorption, otherwise it is called negative adsorption. The adsorption with the most practical application value is positive adsorption, so adsorption can also be defined as the phenomenon in which the concentration of a component at the interface is greater than its enrichment in the bulk phase when the two phases are in contact with each other. Temperature is an important factor affecting adsorption; usually, low temperature will initiate the adsorption reaction to proceed forward: adsorption increases with increasing pressure until saturation is reached. After saturation, no matter what pressure is applied, adsorption will not occur again. Because adsorption is a surface phenomenon, the surface area of the adsorbent will increase the adsorption rate. Liquefied gas is easily absorbed, so attention should be paid to the conditions when adsorbing and separating such gas. The adsorption principle can be divided into the physical type and chemical type [150]. Physical adsorption mainly shows the pore adsorption of porous materials and the secondary interaction between pollutants and functional groups on the adsorbent surface (hydrogen bonding, electrostatic interaction, and hydrophobic interaction); chemical adsorption is mainly the exchange or transfer of electrons between pollutants and adsorbents, resulting in strong chemical bonds [142]. Heavy metal ions (chromium (vi), arsenic (v)) and organic dyes (rhodamine B, malachite green, methylene blue) are common pollutants in water. For nondegradable pollutants, adsorption is one of the methods. Yuan et al. [151] prepared a porous β-cyclodextrin-modified cellulose nanomembrane to enhance the absorption of calcium, phosphorus, and cadmium by bisphenol pollutants in water. Tian et al. [152] prepared cellulose-acetate-nonwoven-membrane-adsorbing heavy metal ions by electrospinning and surface modification of polymethacrylic acid (PMAA) and found that the adsorbed metal ions could be easily desorbed from the membrane surface by using saturated ethylenediaminetetraacetic acid solution, and could be reused for metal ion adsorption. The graphene-embedded nylon nanomembrane prepared by An et al. [153], which could remove pollutants by adsorbing methyl bromide molecules on the surface of graphene sheets, had a strong adsorption capacity.

**Figure 13:**
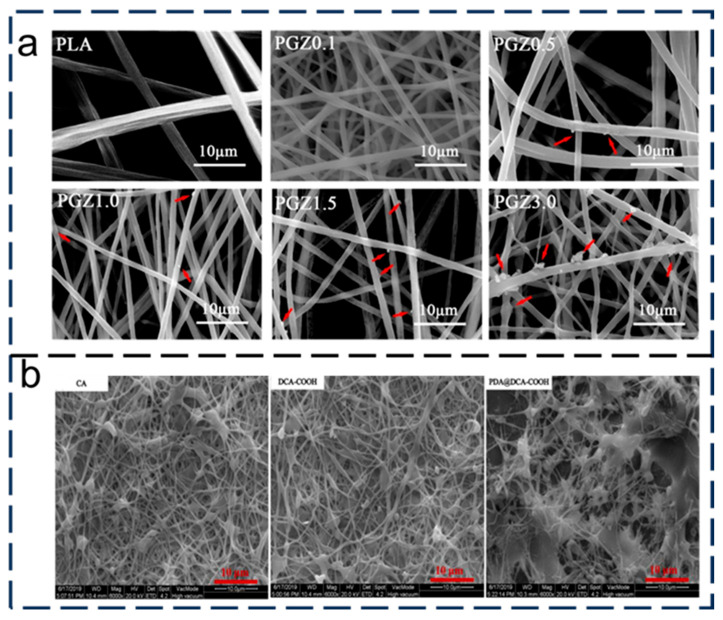
(**a**) SEM images of PLA and PLA/ZIF-8@GO membranes. Reprinted from [147], with open access license. (**b**) FTIR spectra and SEM images of various CA membranes. Reprinted from [155], with open access license.

Electrospun membranes have hydrophilic and hydrophobic membranes, and some membranes have strong adsorbability. Akduman et al. [154] prepared electrospun nanomembranes with TPU and hydrophilic polyvinyl alcohol, and found that due to the hydrophobic structure of TPU nanomembranes, they showed rather low adsorption performance, while BTCA cross-linked polyvinyl alcohol nanomembranes showed good adsorption performance for reactive red 141 dye. Chen et al. [155] prepared a new type of cost-effective multifunctional cellulose acetate membrane by electrospinning (Figure 13b), deacetylation, carboxymethylation, and polydopamine (PDA) coating. The excellent adsorption performance of the composite membrane was due to the strong electrostatic attraction produced by the rich functional groups on the PDA surface. Niu et al. [156] found that HRP successfully coated in situ (PLGA)/PEO- PPO-PEO(F108) electrospun membranes (EFMs) by emulsion electrospinning. The adsorption kinetics of PCP on the electromigration membrane conformed to the quasi-second-order model, and the adsorption capacity was as high as 44.69 mg g^−1^. The interaction between PCP and HRP could significantly improve the adsorption capacity of PCP. Dai et al. [147] prepared a polylactic acid electrospun membrane (Figure 13a) with zeolite imidazole skeleton/graphene oxide hybrid (ZIF-8@GO) as a carrier by the electrospinning method, and found that the degradable polylactic acid/ZIF-8@GO electrospun membrane had higher tensile strength than pure polylactic acid, and the composite membrane also showed good removal ability by adsorption and photocatalytic degradation. Wang et al. [157] showed that the highly porous calcium nanomembrane possesses effective performance for the adsorption of dye solution (rhodamine B aqueous solution), and the silver-loaded highly porous nanomembrane has remarkable antibacterial performance. Silver-loaded porous calcium nanomembrane is an ideal choice for dye wastewater treatment. Through continuous research and development, the method for preparing nanomembranes with enhanced adsorption capacity of micropollutants has been determined. Andrea et al. [158] proved that the ability of cyclodextrin electrospun polyether sulfone nanomembranes to form host–guest complexes with estradiol (E2) and the pesticide chlorpyrifos (CP) was enhanced and the adsorption ability was enhanced. Jiang et al. [159] tested the adsorption of boron nitridepolyacrylonitrile-ethylene oxide nanomembranes on water pollutants such as Congo red, basic yellow 1, and rhodamine B, and their absorption rates were 46%, 53%, and 45%, respectively. Moreover, due to the high heat resistance of 3D BN, these dyes and pollutants can be eliminated and removed from water by heating.

### 5.3. Filtering

Filtration is the operation of separating solids from fluids (liquids and gases) by adding media, which includes mechanical, physical, and biological operations. Suspended toxic particles in water are another threat to human health. To eliminate particle pollution, filtration is a commonly used method. Traditional filter media have a better filtering effect on large particles, but they are not good at removing small-sized particles. It is recognized from a large number of studies that electro-spun nanomembranes have better filtration performance than traditional media and can even remove some ions and molecules. Zhang et al. [160] investigated the filtration performance of electrospun polyimide (PI) nanofibers for PM removal. They fabricated an air filter comprising PI nanofibers supported on a copper mesh, which exhibited excellent capture/adsorption of PM particles. Under a PM index exceeding 300, the filter maintained removal efficiencies of 97–99% and 99–100% for PM_2.5_ and PM_10_, respectively, for more than 120 h of continuous operation. The authors also examined the use of these PI nanofiber filters for automotive exhaust treatment, demonstrating effective removal of particles ranging from <0.3 µm to >10 µm.

**Figure 14:**
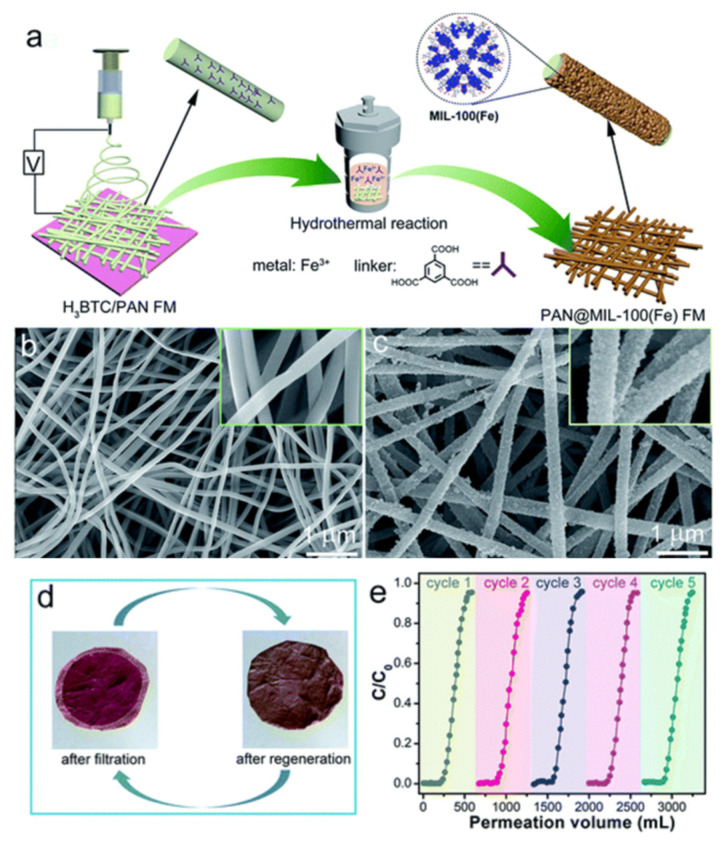
(**a**) Schematic illustration of preparation of the PAN@MIL-100(Fe) FM. SEM images of the H3BTC/PAN FM (**b**) and PAN@MIL-100(Fe) FM (**c**). (**d**,**e**) The optical photographs of the PAN@MIL-100(Fe) FM after AR filtration and regeneration. Reprinted from [161], with open access license.

Electrospinning technology can produce composite membranes with high efficiency because of its advantages, such as simple preparation and good properties. The structure of the ultrafiltration membrane is a kind of microporous filtration membrane. The removal of organic pollutants depends on the geometry of the molecule and the chemical properties of the molecule. We can modify the surface of the ultrafiltration membrane and improve the wastewater removal ability by improving the anti-pollution performance of the material. We can also deal with water pollution through some filtration. Zhao et al. [161] proposed a kind of MIL-100(Fe) porous metal–organic framework, which was uniformly grown on an electrospun polyacrylonitrile membrane by electrostatic spinning and a hydrothermal reaction. The preparation procedure for the MIL-100(Fe) grown on the PAN electrospun fiber membrane (PAN@MIL-100(Fe) FM) is illustrated in Figure 14a. During the hydrothermal reaction, trimesic acid from the H3BTC/PAN FM acted as the nucleation site for in situ growth of MIL-100(Fe) on electrospun PAN fibers. The morphology of the obtained fiber membranes was observed from SEM images and the results are shown in Figure 14b,c. The rough surface was constructed on the electrospun membrane, and the membrane was endowed with superoleophobicity and oil stain resistance under water. Soybean oil could be removed from the oil/water mixture with 99.8% separation efficiency by argon filtration. Because of the binding ability of MIL-100(Fe), PAN@MIL-100(Fe) FM can remove the food additives amaranth red (AR) and vanillic aldehyde (VA) through membrane filtration. The breakthrough volumes for AR and VA by using 3.1 cm^2^ (thickness = 120 μm) of PAN@MIL-100(Fe) FM are 221 mL and 172 mL (pollutant concentration decreases to 0.1 mg L^−1^ from 10 mg L^−1^) (Figure 14). Moreover, the recyclability of the PAN@MIL 100(Fe) FM for AR filtration was also studied. Figure 14e demonstrate its excellent filtration performance.

Therefore, filtration is a good method for some granular pollutants. It can make contact with the substances to be removed with the filter and separate them.

In contaminant removal applications, incorporating functional components into electrospun membranes can assist in targeting specific pollutants through adsorption, degradation, or size exclusion. Factors such as membrane charge, surface chemistry, and internal porosity affect the affinity and retention of various solutes. The distribution of active sites and the accessibility of mass transfer pathways contribute to overall removal behavior.

## 6. Other Critical Applications

Recent years have witnessed growing global attention to environmental protection. A green, safe, and sustainable environment is essential for human survival. As water resources are indispensable for human existence and development, water treatment technologies remain a focal point of research. Below, we introduce electrospun nanofiber membranes for water purification and sensors in aquatic environments.

### 6.1. Antibacterial Water Purification

**Figure 15:**
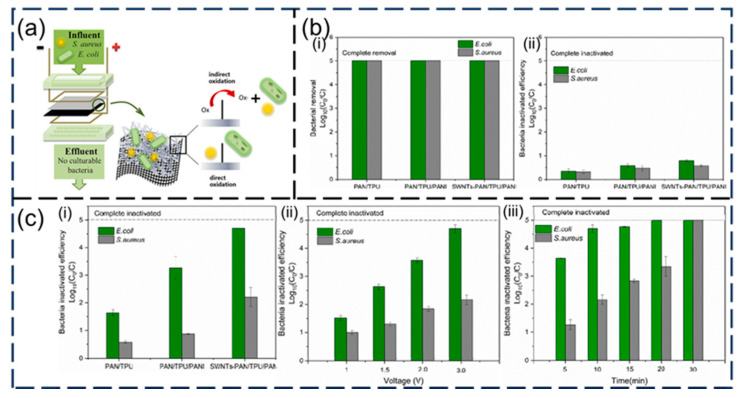
(**a**) Antibacterial mechanism schematic diagram of electrochemical disinfection for fiber membranes. (**b**) Filtration interception (**b**-**i**) and inactivation (**b**-**ii**) performance of different composite membranes. (**c**-**i**) The electrochemical inactivation performances of the three composite membranes (**c**-**ii**) at different potentials (SPTP membrane at 1.0, 1.5, 2.0, and 3.0 V) (**c**-**iii**) and electrolyzed times (SPTP membrane 5, 10, 15, 20, 30 min). Reprinted from [163], with open access license.

Waterborne pathogens pose severe threats to drinking water in developing countries. Research indicates that carbon nanotubes (CNTs) possess advantages such as high disinfection efficiency and stable chemical properties [162]. Based on the disinfection and electrochemical characteristics of CNTs, Xie et al. [163] proposed a single-walled carbon nanotube–polyacrylonitrile/thermoplastic polyurethane/polyaniline (SWNT-PAN/TPU/PANI, SPTP) composite nanofiber membrane prepared via electrostatic spinning technology. By integrating electrochemical and mechanical filtration methods (Figure 15a), the membrane effectively achieves removal and inactivation of bacteria in drinking water. Studies show that the membrane completely removes bacteria through sieving mechanisms under non-electrolytic conditions (Figure 15b). When a 3.0 V voltage is applied, it achieves 5-log bacterial inactivation within 20 min while exhibiting good durability and anti-toxic controlled-release performance (Figure 15c). This is primarily mediated by direct oxidation and ROS-mediated oxidation mechanisms, resulting in persistent and efficient antibacterial properties. The technology not only enhances the electrochemical stability of CNTs but also effectively reduces environmental toxicological risks, providing a novel strategy for drinking water safety.

### 6.2. Sensors for Water Treatment

**Figure 16:**
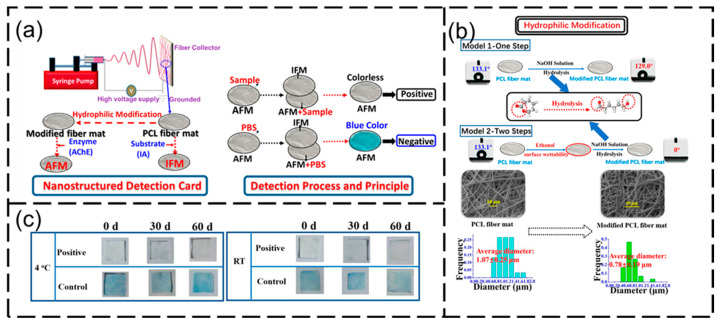
(**a**) Preparation process and pesticide determination principle of the nano-/microstructured detection card. (**b**) Two models used for the hydrophilic modification of PCL fiber mats and the properties of plain and modified fiber mats (n = 50). (**c**) Storage stability of the detection card under 4 °C and at room temperature (RT). Reprinted from [164] with open access license.

The electrostatic-spun nanofiber membrane possesses high porosity and a large specific surface area, among which functional metal-modified nanofiber membranes, due to their abundant active sites, chemical stability, and excellent sensing performance, are widely used for detecting water pollutants, such as pesticides in aquatic environments. Feng et al. [164] used electrostatic spinning technology to encapsulate enzymes (AChE) and substrates (indoxyl acetate, IA) into electrospun nanofibers and modified PCL for hydrophilicity, enhancing enzyme absorption on the fiber membrane and developing a pesticide detection card (Figure 16). Compared to traditional methods, this electrospun membrane offers better stability and lower detection concentrations; relative to national standard values, the minimum detectable concentrations for carbofuran, malathion, and dichlorvos were reduced by 5-fold, 2-fold, and 1.5-fold, respectively.

## 7. Conclusions

This article introduces the application of two-dimensional separation membranes and three-dimensional porous materials based on electrospinning technology in membrane distillation, oil–water separation, solar evaporation, and pollutant removal. The introduction of different water purification processes showed the high efficiency of electrospun nanofibrous membranes for water treatment. At the same time, due to their high porosity, high specific surface area, flexible morphology, strength, interconnected pore structure, and ability to bind chemicals to the membrane surface, electrospray membranes are widely used in water treatment as microfiltration and ultrafiltration media. The overall performance of electrospun membranes across different water treatment applications is fundamentally determined by the synergy between material chemistry and structural design. Surface engineering strategies such as nanoparticle loading, hydrophobic coatings, and asymmetric wettability can significantly modulate wettability, surface energy, and interfacial interactions. Simultaneously, key structural parameters, including fiber diameter, porosity, membrane thickness, and hierarchical architecture, govern mass transfer, thermal insulation, and mechanical strength. Understanding these structure–property relationships provides a theoretical foundation for the rational design of electrospun membranes with high efficiency, durability, and adaptability in membrane distillation, oil–water separation, and solar evaporation systems. In addition, with the further improvement of nanotechnology, the application of nanofibers will be further expanded. However, with the continuous development of industry, environmental problems have become more and more serious, and the existing water treatment methods have some problems, such as their low efficiency and high energy consumption. On the contrary, the low energy consumption, simple operation, and low cost of nanofibrous membranes in the water treatment process will give electrospinning technology great potential in water treatment. However, in practical applications, the surface of some nanofibrous materials is easily affected by external factors. For example, the flux reduction caused by membrane fouling and the instability of the membrane surface are problems. Therefore, it is very important to prepare stable and durable nanofiber membranes. The research into electrospun nanofibrous materials in water treatment still needs to solve many problems, such as making ultrafine nanofibers, enhancing the ability of composite membranes to combine with the nanofiber membrane matrix, and adjusting the membrane pore size to ensure the purity of the membrane. Therefore, the excellent properties of nanofiber materials give them broad application prospects in water treatment.

## Figures and Tables

**Table 1:** Study on parameters of electrospun nanofibers.

Sample	Voltage (kV)	Distance (cm)	Feed (mL/h)	Temperature (°C)	Humidity (%)	Citation
CP-GP	20	15	1.0	25		3
PAN	14		1.0	20	15	15
PEO	5.5		1.0	20	4–10	47
PS	15	12		25	13	51
PLLA	20	16	0.8	21–27	8–10	56
PAN	25	20	1.0	25	14	92
COF-DhaTab/PAN	20	15	1.0	23 ± 5	3	101
CNTSX@PANEN	18	18	2.0		14	103
PLA/CNTS	20	18	0.35		10	106
PMMA-b-P4VP	14		0.2		20	107
PS	10	15	0.5	25	8	108
RC	15		0.5		15	114
PAN	15	14	1.0	30	14	121
ink/PLA	12	15	0.8		15	123
GO/PVA	15	15	0.7	25 ± 1	6	129
PAN	22.5	20	1.5	25	1.5	133
GF	16	10	2.5	25	20	139
SiO_2_@PVA		15			8	152
PLA/ZIF-8@GO	22		0.5	10		153
PVDF/TiO_2_	25	15	4.8	13.5		154
PAN	12.9	20	1.0	20	12	165

## Data Availability

All data generated or analyzed during this study are included in this published article.
